# The Keeley Cure

**Published:** 1893-04

**Authors:** 


ARTICLE VI.

### THE KEELEY CURE.

We clip from the *Medical News* an article published by Dr. Chapman in the *Chicago Medical Recorder* of February. Dr. Chapman states that for the purpose of investigating the "cure" he obtained a position in one of the Keeley Sanitariums and carefully studied out the subject. His views as to the nature of the remedies, gained from personal experience in about three hundred cases, both in and out of the aforesaid sanitarium, are as follows:

The formulary of the gold treatment is almost, if not quite, the same in all of these institutes.

No 1. Tonic. Known in the institutes as the "dope :"

| | |
|---|---|
| R.—Aurii et sodii chlorid, - | gr. xij. |
| Strychninæ nitr., - - | gr. j. |
| Atropinæ sulph., - - | gr. 1-4. |
| Ammonii muriat., - - | gr. vj. |
| Aloin, - - - | gr. j. |
| Hydrastinin, - - - | gr. ij. |
| Glycerini, - - - | ℥j. |
| Ext. fl. cinchon. comp. - | ℥iij. |
| Ext. fl. coca erythrox., - . | ℥j. |
| Aquæ dest., - - - | ℥j.—M. |

S.—1 dram at 7, 9, 11 A. M., at 1, 3, 5, 7, 9 P. M.

No. 2. The injection known in the institutes as the "shot."

| | |
|---|---|
| R.—Strychninæ nitr. - | gr. 9 1-10. |
| Aquæ dest. - - | ad ℥iv. |
| Potass. permangan. q. s. to color. | —M. |

S.—Begie with gtt. 5, which equal gr. 1-40, increase one drop at each injection until the physiologic effect is produced. Four hypodermatic injections to be given daily, beginning at 8 A. M., then at 12 M., 4 P. M., and 8 P. M.

No. 3. Used with No 2:

| | |
|---|---|
| R.—Aurii et sodii chlorid. - | gr. 2½ |
| Aquæ dest. - - | ad ℥j.—M. |

S.—Gtt. 3, every four hours, in combination with the strychnine solution, for the first four days.

This last prescription is used only for the moral effect, which is produced in the following manner: Five drops of the strychnine solution are drawn into the syringe, and then three drops of the gold solution are drawn in and mixed. This produces a golden-yellow color, to which attention is called, and the patient is further assured as to the reality of the presence of the gold by the stain left on the skin after the hypodermatic needle has been removed.

A positive disgust is in almost, if not in every instance, produced in the following manner: The patient is given a drink of whiskey, then the so-called bichloride of gold solution, really a solution of strychnine, is injected in his arm, but at the same time, and without his knowledge, he receives one-tenth grain of apomorphine. It takes but a comparatively short time for the emetic to produce its effects; more or less violent emesis is produced, and the patient soon associating the in-taking of the whiskey with the subsequent disagreeable and sickening vomiting, acquires a positive disgust for the liquor, and is not able to keep any on his stomach. Now he acknowledges the wonderful power of the hypothetical gold compound, and surrenders unconditionally. He is converted, and from an unbelieving scoffer is changed into a disciple and supporter of the prophet. These are the cases that are the most widely advertised, and that have done the most good for the "Keeley Institute."

It will be observed that the foregoing method is nearly the same as that quoted by us in the "Journal" of February 25th. This similarity in the testimony received from different observers inclines us to believe that both the principles on which the cure is based and the therapeutic agents used now are now known to the profession.—*Md. Med. Journal.*

